# Revealing the unexplored fungal communities in deep groundwater of crystalline bedrock fracture zones in Olkiluoto, Finland

**DOI:** 10.3389/fmicb.2015.00573

**Published:** 2015-06-09

**Authors:** Elina Sohlberg, Malin Bomberg, Hanna Miettinen, Mari Nyyssönen, Heikki Salavirta, Minna Vikman, Merja Itävaara

**Affiliations:** VTT Technical Research Centre of FinlandEspoo, Finland

**Keywords:** fungal communities, high-throughput sequencing, crystalline bedrock fracture, Fennoscandian shield, deep biosphere

## Abstract

The diversity and functional role of fungi, one of the ecologically most important groups of eukaryotic microorganisms, remains largely unknown in deep biosphere environments. In this study we investigated fungal communities in packer-isolated bedrock fractures in Olkiluoto, Finland at depths ranging from 296 to 798 m below surface level. DNA- and cDNA-based high-throughput amplicon sequencing analysis of the fungal internal transcribed spacer (ITS) gene markers was used to examine the total fungal diversity and to identify the active members in deep fracture zones at different depths. Results showed that fungi were present in fracture zones at all depths and fungal diversity was higher than expected. Most of the observed fungal sequences belonged to the phylum Ascomycota. Phyla Basidiomycota and Chytridiomycota were only represented as a minor part of the fungal community. Dominating fungal classes in the deep bedrock aquifers were Sordariomycetes, Eurotiomycetes, and Dothideomycetes from the Ascomycota phylum and classes Microbotryomycetes and Tremellomycetes from the Basidiomycota phylum, which are the most frequently detected fungal taxa reported also from deep sea environments. In addition some fungal sequences represented potentially novel fungal species. Active fungi were detected in most of the fracture zones, which proves that fungi are able to maintain cellular activity in these oligotrophic conditions. Possible roles of fungi and their origin in deep bedrock groundwater can only be speculated in the light of current knowledge but some species may be specifically adapted to deep subsurface environment and may play important roles in the utilization and recycling of nutrients and thus sustaining the deep subsurface microbial community.

## Introduction

Fungi are mainly decomposers that play a major role in the biodegradation of plant materials in terrestrial ecosystems. In deep biosphere environments, however, fungal diversity, and their role in ecosystem functioning remains largely unknown. According to the small number of studies conducted thus far viable fungi have been detected in different sub-seafloor and subterranean environments, such as groundwater aquifers, continental sedimentary and hard rocks, and deep subseafloor sediments (Sinclair and Ghiorse, 1989; Madsen and Ghiorse, 1993; Fredrickson and Onstott, 1996; Palumbo et al., 1996; Raghukumar and Raghukumar, 1998; Ludvigsen et al., 1999). Based on a recent review by Nagano and Nagahama (2012) deep sea extreme environments harbor diverse fungal communities. These fungi represent mainly Ascomycota phyla with Eurotiomycetes, Dothideomycetes, Sordariomycetes, and Saccharomycetes being the most abundant fungal classes but also fungi belonging to Basidiomycota and Chytridiomycota have been detected with culture-independed methods. The first viable fungi isolated from deep continental hard rock environments originated from deep crystalline bedrock aquifers in Äspö, Sweden (Pedersen, 1987) and later several yeast species were detected also with DNA-based methods (Pedersen et al., 1996).

Physiological properties of fungi isolated with traditional cultivation-based methods from deep crystalline bedrock fractures indicate that they are adapted to and capable of growing in the subterranean environment (Ekendahl et al., 2003). Identification of facultative anaerobic or strictly anaerobic fungi from deep sea environments indicates that anaerobic conditions are not a limiting factor for fungal growth (Cathrine and Raghukumar, 2009; Jebaraj et al., 2010; Raghukumar, 2012). Remains of bacterial biofilms in these environments also suggest that the biofilms may have supported the nutritious demands of the fungal cells in otherwise extremely oligotrophic environments (Gadd, 2006). Fungi generally prefer mono- or polysaccharides as carbon and energy sources, which would have been provided by the bacterial biofilms. In addition, fungi may be involved in the formation of humic aggregates and carbon contribution by fungal biomass as well as production of extracellular enzymes involved in the cycling of nutrients, as suggested by Raghukumar et al. (2010).

Cultivation-based techniques reveal only a small part of the fungal communities in any environment and with these methods activity of fungi in deep subsurface environments cannot be determined. Novel sequencing technologies would have great potential for obtaining new information on the diversity and ecological role of fungi in the deep geosphere. However, in contrast to the characterization of the bacterial and archaeal communities of deep subsurface habitats, the fungal communities in deep crystalline bedrock fractures have not been characterized by modern culture-independent methods, such as high throughput amplicon sequencing. In this study DNA- and cDNA-based high-throughput amplicon sequencing analysis of the fungal internal transcribed spacer (ITS) gene markers was used to examine the total fungal diversity and to identify the active members of the fungal communities in deep bedrock fracture zones at different depths in Olkiluoto, Finland. The results reveal previously unexplored fungal communities in deep groundwater of crystalline rock fracture zones.

## Materials and methods

### Site description and sampling

Olkiluoto is an island situated in the western coast of Finland. The bedrock of Olkiluoto belongs to the Fennoscandian Shield and consists mostly of Precambrian highly deformed and metamorphosed migmatitic mica gneisses. The characteristics of the site have been described in more detail by Pitkänen et al. (2004), Posiva (2013), Nyyssönen et al. (2012) and Bomberg et al. (2015). In brief, the groundwater in Olkiluoto is anaerobic and saline and salinity increases with depth from 0.1 g L^−1^ at ground level to 100 g L^−1^ at 900 m. The temperature of the groundwater varies from ca. 7°C at 50 m to 20°C at 1000 m and pH is slightly alkaline in all fracture zones. Sulfate is enriched in the upper 300 m and beneath this depth zone, only traces of sulfate are observed. The concentration of methane increases with depth from 300 m.

Altogether, deep groundwater samples from 17 different boreholes at depths ranging from 296 to 798 m were collected between December 14th, 2009 and August 21st, 2013 from the Olkiluoto island in Finland (Table 1). Sampling was done as described in Bomberg et al. (2015). In short, the samples were collected from multi-packered boreholes as well as from open boreholes where the sampling section was packered-off in order to seal off a specific water-conducting fracture zone from the rest of the borehole. This isolated fracture zone was purged by pumping out the water collected between the packers and allowing water from the isolated fracture zone to run into the packered off section of the borehole. In order to assure that sample water was coming only from fracture zones, the packer-sealed fracture zones were pumped for at least 4 weeks before sampling. The conductivity and pH of the pumped water was followed, and when the values settled, it was assumed that the water represents the endemic fracture zone water. Microbial biomass for nucleic acid analyses was concentrated from 500 to 1000 mL samples by filtration on cellulose acetate filters (0.2 μm pore size, Corning) by vacuum suction in the anaerobic chamber. The filters were immediately cut out from the filtration funnels with sterile scalpels and frozen on dry ice.

**Table 1:** **Selected hydrogeochemical characteristics of the Olkiluoto fracture zones investigated in this study**.

Sample^*^	**Ec (ms/m)**	**pH**	**TDS (mg L^−1^)**	**N tot (mg L^−1^)**	**NPOC (mgC L^−1^)**	**DIC (mgC L^−1^)**	**HCO_3_(mg L^−1^)**
OL-KR13/296m_10	897	7.9	4994	0.71	10	27	134
OL-KR13/296m_12	807	7.8	4481	4.4	38	28	116
OL-KR3/303m_12	987	8	5378	0.6	7.8	4.3	26
OL-KR20/323m_13	1116	7.7	6242	0.91	11	13	67
OL-KR6/328m_10	1832	7.9	10670	<0.05	<2.40	4.1	22.6
OL-KR6/330m_13	1800	8	10590	0.051	<2.40	4.6	27
OL-KR25/330m_11	642	7.9	3502	0.96	13	33	171
OL-KR3/340m_11	1012	8.3	5656	1.1	12	4.1	25
OL-KR23/347m_09	2190	7.5	12710	0.42	5.1	3.9	17.1
OL-KR46/372m_13	1778	7.7	10460	1.5	18	5.9	32
OL-KR46/390m_13	1701	7.7	10370	0.86	4.7	18	98
OL-KR5/405m_12	2170	8.1	12880	1.2	19	<3	16
OL-KR49/415m_09	2670	8.1	15900	0.16	<3	<3	9.8
OL-KR9/423m_11	2300	7.5	13430	0.38	5.1	3	11.6
OL-KR9/510m_11	2960	8.1	18580	0.66	6.6	<3	7.3
OL-KR2/559m_10	4110	8.6	25500	1.1	11	<3.75	17.7
OL-KR1/572m_10	3770	7.8	23260	0.41	5	<3.75	14
OL-KR44/693m_13	5520	7.3	37410	10	110	6.5	30
OL-KR29/798m_10	7820	7.3	53210	3.1	<12	<12	7.9

* *Vertical depth and sampling year are presented in the sample name*.

### Geochemical analyses of the groundwater

Conductivity, pH, total dissolved solids (TDS), alkalinity, total organic, and dissolved inorganic carbon and different cations and anions were analyzed from the sampled groundwater. Analysis methods are described before (Posiva, 2013; Bomberg et al., 2015). All analyses were conducted by Posiva Oy (Olkiluoto, Finland).

### Nucleic acid isolation

Total DNA was isolated directly from the frozen cellulose-acetate filters. The filters were cut to pieces with sterile scalpels in a laminar flow hood, and the DNA was extracted and purified with the PowerSoil DNA extraction kit (MoBio Laboratories, Inc., Solana Beach, CA). The isolation was performed according to the instructions of the manufacturer. The isolated and purified DNA was then stored frozen at −80°C until use. Total RNA was isolated directly from the frozen cellulose-acetate filter with the PowerWater RNA isolation kit (MoBio Laboratories, Inc., Solana Beach, CA). The filters were thawed on ice and care was taken to minimize the time of thawing. The intact filters were inserted into the bead tubes with flame-sterilized forceps and the RNA extraction was performed according to the manufacturer's instructions. Negative DNA and RNA isolation controls were also included. DNA contamination of the RNA extracts was checked by PCR with bacterial 16S rRNA gene specific primers 8F (Edwards et al., 1989) and P2 (Muyzer et al., 1993). If no PCR product was obtained, sample was assumed uncontaminated and the RNA extract was submitted to cDNA synthesis. If a PCR product was obtained, the RNA extract was first treated with DNase (Promega, Madison, WI) according to the manufacturer's instructions before cDNA synthesis. Aliquots of 11.5 μL of RNA was incubated together with 250 ng random hexamers (Promega, Madison, WI) and 0.83 mM final concentration dNTP (Thermo Fisher Scientific, Vantaa, Finland) at 65°C for 5 min and cooled on ice for 1 min. The cDNA was synthesized with the Superscript III kit (Invitrogen), by adding 4 μL 5 × First strand buffer, 40 U DTT and 200 U Superscript III to the cooled reactions. To protect the RNA template from degradation, 40 U recombinant RNase inhibitor, RNaseOut (Promega, Madison, WI), was used. The reactions were incubated at 25°C for 5 min, at 50°C for 1 h and at 70°C for 15 min. Two to four parallel reactions per sample as well as no template controls were performed. The parallel reactions were subsequently pooled. RT-PCR was also performed on the negative RNA extraction controls as well as negative reagent RT-PCR controls.

### Amplification library preparation

The amplification libraries for 454 high throughput sequencing were prepared by PCR from the DNA and cDNA samples. Fungal ITS fragments were amplified in a two-step PCR. First, a 420–825 bp long fragment was amplified with primers ITS1F and ITS4 (White et al., 1990; Gardes and Bruns, 1993). Length of the ITS region varies between species (Manter and Vivanco, 2007). The product of this PCR was used as template in a secondary PCR with tagged primers ITS1F and ITS2 (Buée et al., 2009) generating a ca. 400 bp product. First step of PCR amplification was performed in 10 μL and second step in 50 μL reactions containing 1x KAPA Fidelity buffer (Kapa Biosystems, Cape Town, South Africa) (2 mM MgCl_2_), 0.3 mM final concentration of dNTP, 6 pmol of each primer in 10 μL reaction and 25 pmol in 50 μL reaction, 1 unit of KAPA Hifi polyeraze enzyme (Kapa Biosystems, Cape Town, South Africa) and 1 μL of template. The PCR program for both PCR steps consisted of an initial denaturation step at 98°C for 5 min, 39 cycles of 20 s at 98°C, 50 s at 50°C, and 30 s at 72°C. A final elongation step of 5 min was performed at 72°C. In addition negative reagent PCR controls with only PCR-grade water as template were performed to rule out possible contamination. PCR products were confirmed in 1 × SYBR safe-stained 1% agarose gel electrophoresis. The pyrotag libraries were sent for sequencing to Beckman Coulter Genomics (Danvers, MA, USA) where amplicon libraries were purified and smallest and largest fragments were removed based on fragment analysis. Pyrotaq libraries were run on a Genome Sequencer FLX 454 System according to manufacturer's protocol (454 Life Sciences/Roche Applied Biosystems, Branford, CT, USA).

### Sequence processing and analysis

The sequence reads obtained from the 454 high-throughput sequencing were partly processed with in-house pipeline (Salavirta et al., in press). First, sequences were subjected to quality control using the MOTHUR software version v.1.31.2 (Schloss et al., 2009). During this step, adapters, barcodes, and primers were removed from the sequence reads, and the quality of base-calls was assessed in order to remove erroneous reads from the data set. Subsequently, chimeric sequence reads were removed from the data set with the USEARCH algorithm version 5.2.236 (Edgar, 2010) by *de novo* detection and through similarity searches against the 97% representative OTU set of the UNITE reference database (Kõljalg et al., 2013).

Groups of similar sequences, i.e., Operational Taxonomic Units (OTUs), were selected from the chimera-filtered sequence data set following open-reference OTU-picking protocol of QIIME v. 1.7.0 (Caporaso et al., 2010) against the 97% identity UNITE database OTU sets (Kõljalg et al., 2013). OTU clustering was performed with UCLUST v. 1.2.22q (Edgar, 2010) and the seed sequences were selected as the representative OTU sequences. Only few fungal sequences were amplified from negative control. This is possibly due to two-step PCR and these OTUs based on these sequences were removed from the entire sequence data set. All reads that failed to hit the UNITE reference database with a minimum of 60% identity threshold were discarded as sequencing error. Next, singleton OTUs, i.e., OTUs that were represented by a single sequence, were filtered from the data set. Finally, taxonomy from domain to species-level was assigned to OTUs via representative OTU sequences with BLASTN with a maximum *E*-value of 0.001 (Altschul et al., 1990). Alpha diversity indexes chao1 (Chao, 1984) and Shannon diversity index (Shannon, 1948) were calculated from normalized sequence data where sequence data was subsampled to 1500 sequences to adjust for sequencing coverage. Heatmaps of the fungal communities were generated in the R environment (R Development Core Team, 2008) utilizing the reshape2 (Wickham, 2007), grid (Murrell, 2005), and ggplot2 (Wickham, 2009) packages.

### Statistical analysis

Non-metric multidimensional scaling analyses (NMDS) for comparing the similarity of the fungal communities at class level between the different samples and the effect of chemical parameters on the microbial communities was performed using the PAleontological STatistics (PAST) program (Hammer et al., 2001). Non-Euclidean Bray-Curtis distance matrix was generated with PAST and correlation coefficient values of the matrix was calculated with 1000 permutations with R. In addition Pearson's correlation between total (DNA fraction) and active (RNA fraction) fungal communities was calculated at genus level with compare_taxa_summaries.py command within QIIME. Venn diagrams of each sample were calculated with MOTHUR showing shared OTUs between DNA and RNA fraction.

### Accession numbers

The fungal ITS gene region sequences have been submitted to the European Nucleotide Archive (ENA, https://www.ebi.ac.uk/ena/) under accession numbers ERS706390- ERS706426.

## Results

### Fungal diversity and community structure in different bore holes

**Figure 1:**
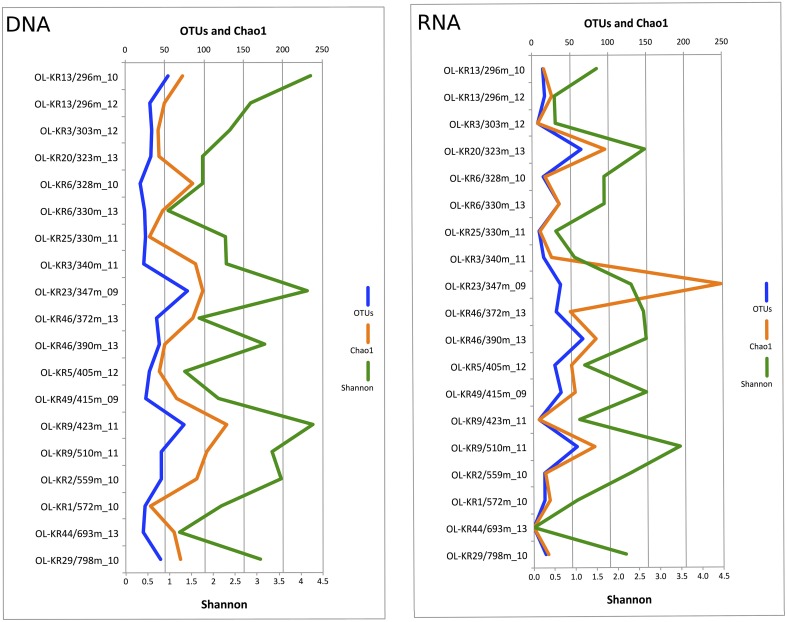
**Fungal diversity in deep groundwater of crystalline bedrock fracture zones in Olkiluoto, Finland**. Number of observed taxonomic units (OTUs), estimated number of OTUs (Chao1) and Shannon diversity index achieved from the sequence data subsampled to 1500 sequences are presented.

Fungal sequences were detected in the DNA fraction of all of the 19 analyzed fracture water samples and in 18 samples of the RNA fraction. In total 378,831 quality-filtered fungal ITS sequences were obtained from the different fracture zones. The number of obtained sequences ranged from 306 to 24,616 in different samples with a median of 10,941 sequences per sample (Table S1). When comparing the Chao1 OTU richness estimate values to true detected OTU numbers, 22–100% of estimated fungal OTUs were obtained from the subsampled sequence data meaning that sequencing depth was sufficient enough to fully characterize the fungal communities in most of the samples. Altogether, 965 fungal OTUs ranging from 33 OTUs at 328 m in OL-KR6 to 163 OTUs in OL-KR9 at 423 m in DNA fraction and 7 OTUs in OL-KR3 at 303 m to 69 OTUs at 330 m and 405 m in RNA fraction were detected in the total sequence data (Table S1). Fungal diversity based on subsampled OTU richness in the DNA samples originating from the fracture waters peaked at 347 m in OL-KR23 (79 OTUs) and was lowest at 328 m in OL-KR6 (19 OTUs) (Figure 1). In RNA fraction highest OTU richness was detected at 390 m in OL-KR46. However, no clear connection between sampling depth and fungal OTU numbers was detected. In the DNA fraction highest Shannon diversity index (H′ = 4.3), which indicates the abundance and evenness of the species present, was obtained at 423 m depth in sample OL-KR9 and lowest at 330 m depth in sample collected from OL-KR6 (H′ = 1.0). In the active fungal community highest diversity was observed at 510 m in OL-KR9 (H′ = 3.5). No fungi were detected in OL-KR44 at 693 m in the active fungal community.

Most of the observed fungal sequences belonged to the phylum Ascomycota (63.9%). Phylum Basidiomycota was represented by 8.9% of all the sequences. Other fungal phyla detected were Chytridiomycota, Glomeromycota, and Zygomycota, which were represented as a minor (0.1–2.1%) part of the whole fungal community. Altogether approximately 25% of the fungal sequences obtained were identified as fungal according to UNITE database but a more specific classification remained unknown. This can be due to insufficient representation of fungal sequences in the sequence databases or these species have not been characterized before and could be considered as novel.

**Figure 2:**
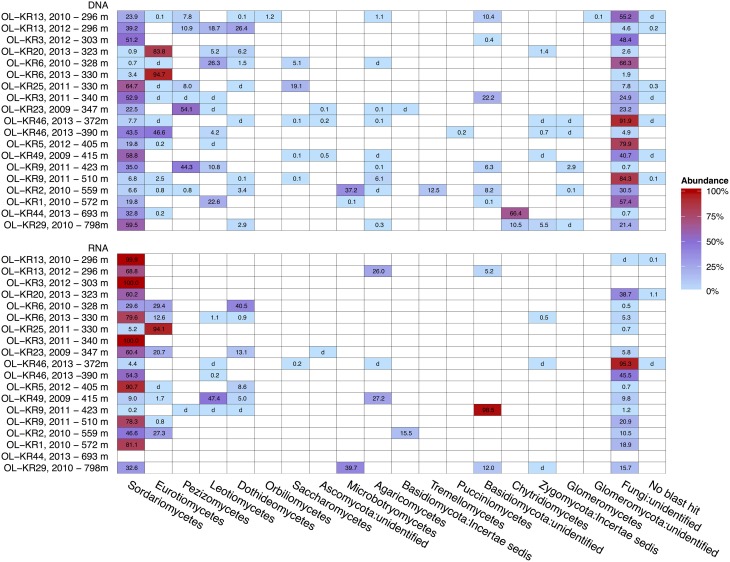
**Heatmap of fungal taxonomy in deep groundwater of crystalline bedrock fracture zones in Olkiluoto, Finland**. Taxonomic classification of the fungal sequence reads obtained by high throughput sequencing of the total (DNA) and active (RNA) fungal communities presented at the class-level. The samples are arranged by true vertical depth from the surface down. d, detected; but relative abundance less than 0.1%.

Structure of the fungal communities varied between different fracture zones and sampling times (Figure 2). Sequences affiliating with Ascomycota dominated fungal community in both DNA and RNA fraction in most of the fracture zones. However, Basidiomycota was the dominating phylum in OL-KR2 at 559 m (58%) in the total fungal community and in OL-KR9 at 423 m (98.5%) and OL-KR29 at 798 m in the active fungal communities. In addition, Chytridiomycota was the dominating phylum in OL-KR44 at 693 m (66%) in the total fungal community.

Sordariomycetes from the Ascomycota phylum was the major active fungal class (47–100% of all the sequences) in most of the boreholes and also the dominating fungal class in the total fungal community in OL-KR13 at 296 m, OL-KR3 at 303 m, OL-KR25 at 330 m, OL-KR3 at 340 m, OL-KR49 at 415 m and OL-KR29 at 798 m (39–65%) (Figure 2). OTUs belonging to Sordariomycetes were mostly related to *Nectria* genus and minority to *Fusarium*, *Pochonia, Pseudallescheria*, and unidentified Hypocreales groups (Figure S1). Fungal class Eurotiomycetes was identified as dominating fungal class in total fungal community of OL-KR20 at 323 m (84%) and OL-KR6 at 330m (2013) (95%) and in active fungal community of OL-KR25 at 330 m (94%). The majority of the sequences belonging to the class Eurotiomycetes were members of the *Penicillium* genus. In addition sequences most closely related to genus *Aspergillus* were found from OL-KR46 at 390 m and OL-KR2 at 559 m as a minor group. Other detected Ascomycota fungal classes in Olkiluoto groundwater samples were Dothideomycetes that dominated the active community in OL-KR6 at 328 m (2010) (40,5%), Leotimycetes that dominated the active fungal community in OL-KR49 at 415 m (47%) and total identified fungal community in OL-KR6 at 328 m (2010) (26%) and OL-KR1 at 572 m (23%) and Saccharomycetes in OL-KR25 at 330 m (19%) and OL-KR6 at 328 m (2010) (5%) total community (Figure 2). In addition sequences belonging to class Orbilliomycetes was only found in OL-KR13 at 296 m (2012), but they contributed only as a minor (1.2%) part of the fungal community.

In OL-KR2 at 559 m in the total fungal community and OL-KR29 at 798 m in the active community where Basidiomycota was the dominating identified phylum most of the sequences were closely related to class Microbotryomycetes (37–39%) and more closely to Sporodiobolales order and *Sporobolomyces* and *Rhodotorula* genera (Figure S2). In addition in OL-KR2 at 559 m Tremellomycetes and more specifically *Cryptococcus*-like yeast sequences were detected in total community and Malasseziales order in active community. Other identified Basidiomycota classes in Olkiluoto boreholes were Agaricomycetes in OL-KR13 at 296 m and in OL-KR49 at 415 m in the active community (Figure 2). Pucciniomycetes was detected in OL-KR46 at 390 m as a minor part of the total community (0.2%). The Chytridiomycota phylum dominated the total fungal community in OL-KR44 at 693 m (66%) and was also present in OL-KR29 at 798 m. Chytridiomycota sequences from these boreholes were most similar with order Rhizophydiales. No Chytridiomycota sequences were detected in the active fungal community.

### Statistical analysis of fungal diversity and correlation to geochemistry

**Figure 3:**
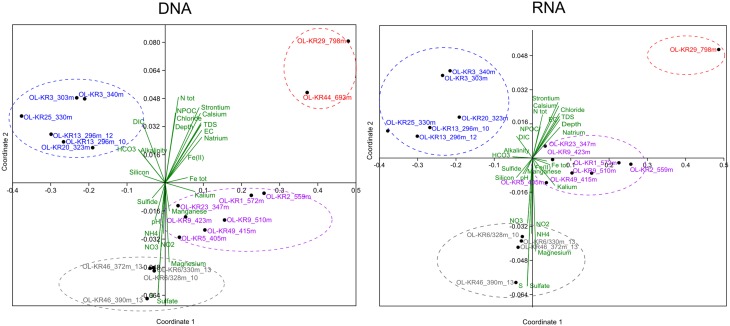
**Fungal community structure in relation to sample depth and its associated environmental factors in the groundwater of Olkiluoto fracture zones**. A non-metric multidimensional scaling analysis of Bray–Curtis distances was performed to examine the relative abundance of fungal OTUs clustering at the class level.

Non-metric multidimensional scaling analysis of fungal communities and environmental parameters grouped the samples into four clusters in both DNA and RNA fractures (Figure 3 and Table S2). The deepest samples (693 and 798 m) clustered together in the DNA fraction that indicates that depth has an influence in the fungal community structure and fungal communities in the deepest communities are most similar. In addition, at greater depth higher salinity also affect the communities and slightly lower pH was observed to significantly correlate with fungal community structure (*p* < 0.05). At depths from 296 to 340 m carbon availability (DIC, HCO_3_) affected the fungal communities in both DNA and RNA fractions and these samples grouped together. In those fracture zones concentrations of DIC and HCO_3_ were higher than in deeper fracture zones ranging from 4.1 to 33 mgC L^−1^ of DIC and 25 to 134 mg L^−1^ of HCO_3_ (Table 1 and Table S3). Amount of total organic carbon was highest at 693 m in OL-KR44 where concentration of NPOC was 110 mgC L^−1^. However, in the NMDS analysis organic carbon concentration did not significantly affect the fungal community structure (*p* > 0.1). Ammonium, nitrate, nitrite, magnesium, and sulfate concentrations were associated with the changes in fungal community profiles especially in OL-KR46 at 372 and 390 m and OL-KR6 at 328 and 330 m in both RNA and DNA fraction that grouped together (Figure 3). Especially nitrite and nitrate had a significant effect on the fungal communities in the DNA fraction (*p* < 0.001), but concentrations are very low.

The similarities of fungal communities between DNA and RNA fractions were assessed at genus level by Pearson's one-sided t-distribution tests, with the hypothesis that positive correlation would be detected between total and active communities. In the total dataset Pearson's correlation was 0.33 (± 0.3 95% CI, *p* < 0.001) between the total and active fungal communities. When we compared specific samples, statistically significant correlation (*p* < 0.05) was found in 10 of the 18 fracture zones (Table S4). Statistically significant Pearson's correlations varied between 0.20 and 0.99 with highest correlation found at 372 m in OL-KR46 and weakest at 303 m OL-KR3. Moderate or strong correlation was found in seven of the fracture zones (0.32–0.99). The number of shared OTUs between DNA and RNA fraction was 0–23% (Figure S3).

## Discussion

Terrestrial deep subsurface mycology is still an unexplored research field as the major research done until now has been focusing on the diversity and functional studies of bacteria and archaea. To our knowledge this is the first study where fungal communities in deep groundwater of crystalline bedrock fracture zones were studied using high-throughput amplicon sequencing. Our results show that diverse and active fungal communities exist in the deep subsurface in Olkiluoto, Finland. Actually, in most of the studied fracture zones the fungal diversity was higher than what has been detected so far in deep sea environment. Deep sea studies have reported up to 43 fungal OTUs with >99% sequence similarity by using fungal ITS region cloning and sequencing (Lai et al., 2007; Nagano et al., 2010; Singh et al., 2012) and Orsi et al. (2013a) detected up to 26 fungal OTUs with 454 pyrosequencing of the eukaryotic 18S rRNA region, whereas we detected up to 163 OTUs in different fracture zones and depths. In our study fungal ITS1 region was chosen over ribosomal genes because it is highly variable and can separate fungi even at species level (Lindahl et al., 2013). ITS2 is considered generally less variable in length than ITS1 and is somewhat better represented in sequence databases. However, ITS1 and ITS2 share many properties, and similar results can be obtained with these two marker genes (Bazzicalupo et al., 2013). With cultivation-based methods five *Rhodotorula* and *Cryptococcus* yeast species and 17 molds have been detected in Fennoscandian rock aquifers in Äspö, Sweden (Ekendahl et al., 2003). Fungi belonging to the Ascomycota phylum were the most abundant in Olkiluoto fracture zones and this is in good agreement with findings from deep sea environments (Nagano and Nagahama, 2012). The deepest fracture zones with higher salinity and temperature and lower pH, were the only ones where fungi belonging to the Chytridiomycota phylum were detected. In addition, the number of basidiomycete species also increased with depth (Figure 2). Altogether 25% of the fungal sequences remained unidentified and some of these OTUs could be potentially novel species that have not been characterized before.

Fungal diversity in the deep crystalline fracture water in Olkiluoto was surprisingly high. Unexpectedly no fungal OTUs were detected in the RNA fraction at 693 m depth, where the highest concentration of total organic carbon that fungi could easily use in their metabolism was detected. Although no clear connection between fungal diversity and some of the geochemical parameters were detected, NMDS analysis showed that salinity, which increases with depth, had an influence on the fungal community structure in deeper fracture zones and also nitrogen compounds, sulfate, and inorganic carbon were associated with the changes in the fungal communities at more shallow depths. Fungi are involved in many biogeochemical cycles such as nitrogen and sulfur cycles and fungi are for example able to solubilize minerals, dissolute, and precipitate metal ions, degrade silicates and dissolve rock phosphates in oxygen-limited environments (Gadd, 2006; Sterflinger, 2010). Some filamentous fungi and yeast species are able to oxidase sulfur and sulfur compounds and release sulfate to environment (Wainwright and Grayston, 1989; Reitner et al., 2006; Sterflinger, 2010). Thus, fungi could provide sulfate to sulfate-reducing bacteria and could potentially be involved in the sulfur cycle in subsurface environment. Although fungi and bacteria are competing for the same low amounts of nutrients in subsurface oligotrophic conditions, they can also benefit from each other. For example, Fournier et al. (1998) found that the yeast *Rhodotorula rubra* has a stimulating effect on the growth of the iron sulfide-oxidizing bacterium *Thiobacillus ferrooxidans*. Similar cooperation between fungi and bacteria could potentially occur also in Olkiluoto deep fracture zones.

Members of the Sordariomycetes were the most commonly observed fungi from Olkiluoto fracture waters. These fungi are ubiquitous and cosmopolitan and function in virtually all ecosystems (Zhang et al., 2006). The group includes pathogens, endophytes of plants, mycoparasites and saprobes involved in decomposition and nutrient cycling but their role in deep biosphere ecosystems is not studied. Sordariomycetes are together with Eurotiomycetes, Saccharomycetes and Dothideomycetes one of the most frequently detected fungal taxa in deep sea environments where living conditions resemble the ones of subterranean deep fracture zones (Nagano and Nagahama, 2012). However, phylotypes within the class Sordariomycetes are few and unique to the studied deep sea areas and their role and functions are still unknown. The fungal sequences obtained from Olkiluoto fracture waters belonging to the Sordariomycetes class were closely related to members of the Nectriaceae family and *Nectria* and *Fusarium* genera. The family Nectriceae includes, e.g., facultative anaerobic microscopic fungi capable of using nitrate or nitrite as alternative terminal electron acceptor in their respiration in the absence of oxygen (Kurakov et al., 2008). Fungal species belonging to *Nectria* have been detected in deep sea sediments (Singh et al., 2012). Some species belonging to genus *Fusarium* that are capable of denitrification have been found in deep sea environments, especially from oxygen-depleted regions (Jebaraj et al., 2010).

NMDS analysis showed correlation between fungal community in OL-KR6 at 330 m, where the most *Penicillium* -sequences were detected, and ammonium, nitrate and nitrite concentrations. This indicates that these species might be involved in nitrogen cycle also in the deep fracture zones in Olkiluoto. Strong correlation between fungal diversity and nitrate has been found also from deep marine sediments (Orsi et al., 2013b) *Penicillium* and *Aspergillus* species are common in outdoor air and terrestrial environments but they are also frequently detected in deep sea environments (Nagano and Nagahama, 2012; Raghukumar, 2012). Deep sea species differed from terrestrial species by their physiological properties and that they were adapted to an aqueous environment (Raghukumar and Raghukumar, 1998; Damare et al., 2006; Damare and Raghukumar, 2008). Salt-tolerant *Penicillium* and *Aspergillus* species have also been identified from oxygen-deficient environments (Raghukumar, 2012) and from anaerobic marine sediments where they were reported to play on important role in the denitrification process (Jebaraj et al., 2010). This suggests a possible versatile role of fungi in major ecological processes in extreme nutrient-poor environments, such as Olkiluoto deep fracture zone fluid. *Aureobasidium* and *Cladosporium* genera found especially in OL-KR6 at 328 m from the RNA-fraction and OL-KR13 at 296 m from the DNA-fraction are reported in many deep sea environment studies (Damare et al., 2008). Common characteristics of these fungal groups are resistance or adaptation to high osmotic pressure that is essential for survival in extreme conditions such as the ones in deep subsurface environments.

The most common classes of the Basidiomycota phylum detected in Olkiluoto groundwater were Microbotryomycetes, Tremellomycetes, and order Malasseziales. These fungal groups are common in deep sea environments (Nagano and Nagahama, 2012) and RNA transcripts from active species within these phyla have been detected in deep marine sediments (Orsi et al., 2013a). Yeast genera within class Microbotryomycetes detected from Olkiluoto were *Rhodotorula* and *Sporobolomycetes* related species. The *Rhodotorula* strain isolated from Fennoscandian rock aquifers in Äspö was able to grow in a wide range of NaCl concentrations (0–100 g L^−1^) and pH interval of 4–10 at temperatures ranging from 4 to 30°C (Ekendahl et al., 2003). Salinity, pH and temperature in Olkiluoto are also within these limits. This indicates that these yeast species are adapted to conditions in the deep subsurface environment. In Olkiluoto *Sporobolomycetes* related species were also detected in the RNA-fraction, which proves that these species were active in these conditions. *Cryptococcus*-like yeasts from class Tremellomycetes that were identified from Olkiluoto have also been detected from other deep igneous rock aquifers sites on the Fennoscandian shield (Ekendahl et al., 2003) and deep sea environments like deep sea methane seeps (Takishita et al., 2006, 2007). *Cryptococcus*-like yeasts were identified from the active community in OL-KR2 at 559 m where also high methane concentration (386 ml L^−1^) was detected (Bomberg et al., 2012) that fungi could potentially use in their metabolism. Yeast genera within the order Malasseziales observed in Olkiluoto fracture zone water were closely related to cultured *Malassezia* sp. LCP-2008 and uncultured *Malassezia* from deep sea sediments (Singh et al., 2012). Phylotypes belonging to *Malassezia* sp. have also been recovered from methane hydrate-bearing deep sea sediments (Lai et al., 2007). These yeast species could potentially be methylotrophic and could play a crucial role in converting methane into more accessible carbon and energy substrates for the use of the microbial community (Lai et al., 2007; Raghukumar et al., 2010). In addition, fungi have been found to be involved in methane release in a coal mine (Beckmann et al., 2011). In the coal mine, weathering of coal, and timber were initiated by fungi and in the lower, oxygen depleted regions fungi were observed to perform incomplete oxidation of coal and wood substrates and release reduced carbon substrates, which can be channeled into methanogenesis.

Chytridiomycota that dominated in DNA-fraction of OL-KR44 at 693 m were most similar to the order Rhizophydiales that also have been found in oxygen-deficient marine environments (Raghukumar, 2012). Chytridiomycota are the earliest diverging lineage of fungi and produces zoospores, which indicates adaptation to aquatic environments (Nagano and Nagahama, 2012; Raghukumar, 2012). Chytridiomycota was not detected in RNA fraction. However, statistically significant correlation of fungal taxonomy profiles between total and active communities was found in majority of the fracture zones, which suggests that fungal communities were similar in both DNA and RNA fraction. In three of these samples only weak correlation and in eight of the fracture zones no significance correlation was found, which indicates that total and active communities in these fracture zones were different. In half of samples with no significant correlation insufficient sampling depth of the RNA fraction may have affected the result. In OL-KR6 at 328 m, OL-KR9 at 423 m, OL-KR49 at 415 m and OL-KR25 at 330 m total and active communities appear to be truly different, suggesting that different species are active in these fracture zones compared to total community.

The universal distribution of the many fungal species detected also in deep subsurface environments raises the question about possible contamination. It is known that controlling contamination during drilling in hard rock is more difficult than for example sedimentary rock and life dwelling in fracture zones is exposed to drill water during drilling. Origin of the fungi in Olkiluoto fracture zones is unknown and possible runoff from the surface or contamination during drilling cannot be ruled out based on this study. On the other hand, fungi can be authentic members of the microbial community in the different fracture zones. In this study water from the drill hole was purged for a long time after drilling and by using packers only water originating from the specific fracture zone was collected. Hydrogeochemical characteristics of the fracture fluids also indicates that the chemical parameters were stabilized confirming that water from specific fracture zone was collected. Most importantly, active fungi were found from Olkiluoto groundwater and this indicates that fungi have adapted to deep biosphere conditions and are able to maintain cellular activity.

The metabolic activities of fungi in deep terrestrial environment remain still unknown. However, the first fungal metabolic transcriptomics study from sub-seafloor environment confirms the previous suggestions of living fungi and active fungal metabolism in the deep marine biosphere (Orsi et al., 2013b). The authors showed that 5% of the obtained transcripts were involved in carbohydrate, amino acid, and lipid metabolism suggesting that fungi have a role in organic carbon cycling in sub-seafloor sediment. Fungal expression of transcripts encoding hydrolases involved in protein, carbohydrate, and lipid degradation suggests that they degrade a variety of organic substrates. Fungal dissimilatory nitrate reductase (nar) transcripts involved in energy production were found, which indicate that fungi are involved in nitrogen cycle, probably reducing nitrate and nitrite resulting from nitrate reduction performed by bacteria.

In our study surprisingly high diversity of active fungi were detected for the first time in deep groundwater of crystalline rock fractures. Unlike bacterial 16S rRNA gene, the RNA fraction of fungal ITS is only present in the cell when the genomic copy is being actively transcribed and thus is a true evidence that fungi are active in the deep fracture waters (Blazewicz et al., 2013). The most interesting question now is what are these fungi doing in the deep fracture zones in Olkiluoto and in deep terrestrial environment in general and what is their role in the whole microbial community? Metatranscriptomic studies could be the answer and next step in understanding the functionality of the fungal communities in deep subterranean environments. Interesting was that the amount of organic carbon did not correlate with fungal diversity and activity in deep fracture zones suggesting that fungi may have some other functions in deep subterranean environments than degradation of organic materials.

### Conflict of interest statement

Posiva Oy funded this research. The authors declare that the research was conducted in the absence of any commercial or financial relationships that could be construed as a potential conflict of interest.
