# Supplementary material for: Revealing the unexplored fungal communities in deep groundwater of crystalline bedrock fracture zones in Olkiluoto, Finland
Source: Front Microbiol. 2015 Jun 9;6:573. doi: 10.3389/fmicb.2015.00573 (PMC4460562; doi:10.3389/fmicb.2015.00573)
Supplement: Supplementary file 1 [file Table1.PDF]

Table S1. General sequence statistics of fungal ITS sequences in the samples. OTUs are reported at 97% identity and the alpha diversity of the sequence data is estimated from 1500 randomly sampled sequences/sample

		Total sequence data					Normalized sequence data			
Sample	Type	Sequences	OTUs (97%)	Estimated number of OTUs chao1	% diversity obtained	Shannon	OTU count	Estimated number of OTUs chao1	% diversity obtained	Shannon
OL-KR13/296m_10	DNA	11,141	89	92	96.7	4.3	54	73	73.8	4.2
	RNA	3,145	17	25	69.4	1.6	14	16	87.5	1.5
OL-KR13/296m_12	DNA	10,941	60	69	86.8	2.8	31	49	62.8	2.9
	RNA	14,082	37	50	73.7	0.5	17	26	64.6	0.5
OL-KR3/303m_12	DNA	6,586	51	53	96.8	2.4	34	42	81.9	2.4
	RNA*	591	7	7	100.0	0.6	7	7	100.0	0.6
OL-KR20/323m_13	DNA	12,496	65	71	92.2	1.8	33	43	77.2	1.8
	RNA*	1,421	64	95	67.2	2.7	64	95	67.2	2.7
OL-KR6/328m_10	DNA	6,291	33	46	71.7	1.8	19	85	22.4	1.8
	RNA	11,024	35	36	97.7	1.7	15	17	90.0	1.7
OL-KR6/330m_13	DNA	9,470	47	64	73.3	1.0	25	47	53.2	1.0
	RNA*	436	69	91	76.1	4.2	35	36	97.7	1.7
OL-KR25/330m_11	DNA	5,171	42	49	85.1	2.2	26	31	84.8	2.3
	RNA*	306	9	10	90.0	0.5	9	10	90.0	0.5
OL-KR3/340m_11	DNA	11,402	50	71	70.1	2.3	23	89	25.8	2.3
	RNA	6,745	23	23	100.0	1.0	15	26	58.8	1.0
OL-KR23/347m_09	DNA	14,089	148	152	97.6	4.2	79	99	80.2	4.2
	RNA	15,422	61	66	93.0	2.3	37	247	15.0	2.3
OL-KR46/372m_13	DNA	17,031	80	91	87.7	1.7	40	86	46.5	1.7
	RNA	6,273	66	91	72.5	2.7	31	49	62.8	2.6
OL-KR46/390m_13	DNA	15,211	82	91	90.6	3.2	43	49	87.0	3.2
	RNA	1,582	65	83	77.9	2.7	65	83	77.9	2.7
OL-KR5/405m_12	DNA	4,927	41	46	90.1	1.3	30	43	69.8	1.3
	RNA	19,755	69	71	97.0	1.2	29	50	58.0	1.2
OL-KR49/415m_09	DNA	17,883	60	72	83.6	2.1	26	65	40.0	2.1
	RNA	8,715	62	64	96.4	2.7	37	55	67.0	2.7
OL-KR9/423m_11	DNA	13,481	163	181	90.1	4.4	74	128	57.6	4.3
	RNA	24,616	23	26	88.5	1.1	7	8	93.3	1.1
OL-KR9/510m_11	DNA	23,537	111	121	91.7	3.4	45	103	43.8	3.3
	RNA*	516	57	80	70.9	3.5	57	80	70.9	3.5
OL-KR2/559m_10	DNA	8,820	94	145	64.8	3.6	45	90	49.8	3.5
	RNA	7,569	24	28	86.5	2.3	14	16	87.5	2.3
OL-KR1/572m_10	DNA	13,992	52	57	92.0	2.2	24	31	76.9	2.2
	RNA	18,556	27	30	90.0	1.0	15	22	68.2	1.0
OL-KR44/693m_13	DNA	7,657	50	60	82.7	1.2	22	61	36.1	1.2
	RNA**									
OL-KR29/798m_10	DNA	15,268	90	101	89.0	3.1	44	70	63.3	3.1
	RNA	12,683	34	40	84.1	2.2	16	19	82.8	2.2

* These samples had less than 1,500 sequences and only total sequence data is presented

** no fungal ITS product achieved with PCR, below detection limit?
