# Supplementary material for: Revealing the unexplored fungal communities in deep groundwater of crystalline bedrock fracture zones in Olkiluoto, Finland
Source: Front Microbiol. 2015 Jun 9;6:573. doi: 10.3389/fmicb.2015.00573 (PMC4460562; doi:10.3389/fmicb.2015.00573)
Supplement: Supplementary file 2 [file Table2.PDF]

Table S2. Dimension coordinates and correlation coefficients for the non-metric multidimensional scaling analysis. The p values are based on 1000 permutations. Statistically significant variables are indicated in bold and have been marked with the following significance codes: 0 '***' 0.001 '**' 0.01 '*' 0.05 '.'.

	DNA					RNA			
	**NMDS1**	**NMDS2**	**r2**	**Pr(>r)**		**NMDS1**	**NMDS2**	**r2**	**Pr(>r)**
Depth	-0.51855	0.85505	0.0699	0.533467		0.97739	-0.21142	0.0367	0.7532
pH	-0.52485	-0.8512	0.3122	0.046953	*	-0.81427	0.58048	0.025	0.8082
EC	-0.55104	0.83448	0.054	0.625375		0.95947	0.2818	0.0168	0.8771
DIC	0.58684	0.8097	0.1465	0.274725		0.62607	-0.77976	0.2195	0.1279
NPOC	0.35034	0.93662	0.2361	0.112887		0.71081	-0.70338	0.0771	0.4286
HCO3	0.7113	0.70289	0.1213	0.341658		0.63061	-0.7761	0.1874	0.1718
TDS	-0.45097	0.89254	0.0548	0.619381		0.99968	0.02512	0.0157	0.8841
Alkalinity	0.71538	0.69874	0.1211	0.342657		0.63215	-0.77485	0.1889	0.1708
Sulfate	0.63269	-0.77441	0.1721	0.214785		0.40542	-0.91413	0.0385	0.7293
S	0.62708	-0.77896	0.1768	0.208791		0.43393	-0.90095	0.0357	0.7423
Sulfide	0.75442	-0.65639	0.1462	0.292707		0.94461	-0.32819	0.0538	0.6104
NH4	0.84616	0.53293	0.0661	0.603397		0.40659	-0.91361	0.13	0.2128
NO3	0.9241	-0.38215	0.7715	0.000999	***	0.79661	-0.6045	0.0711	0.5475
NO2	0.92574	-0.37816	0.7718	0.000999	***	0.79586	-0.60548	0.069	0.5594
N.tot	0.34023	0.94034	0.2853	0.057942	.	0.63633	-0.77142	0.0894	0.3886
Fe.II.	0.29213	0.95638	0.1342	0.2997		0.03439	-0.99941	0.0466	0.6424
Fe.tot	-0.99173	0.12833	0.0955	0.446553		0.60436	0.79671	0.0175	0.8641
Natrium	-0.63657	0.77122	0.0256	0.793207		0.99748	-0.07091	0.0157	0.8901
Kalium	0.76472	0.64436	0.0031	0.98002		0.81133	-0.58458	0.163	0.2368
Calsium	-0.35007	0.93672	0.0959	0.423576		0.93985	0.34159	0.0181	0.8621
Magnesium	0.79569	-0.6057	0.086	0.496503		0.31882	-0.94781	0.1283	0.2517
Manganese	-0.79754	0.60327	0.0061	0.984016		-0.68354	0.72991	0.136	0.2078
Chloride	-0.48269	0.87579	0.0571	0.602398		0.99952	0.03088	0.0136	0.8991
Silicon	0.57573	0.81764	0.2797	0.096903	.	0.80496	-0.59333	0.0847	0.4525
Strontium	-0.26466	0.96434	0.0936	0.423576		0.99964	0.02669	0.0111	0.9131
