# Supplementary material for: Revealing the unexplored fungal communities in deep groundwater of crystalline bedrock fracture zones in Olkiluoto, Finland
Source: Front Microbiol. 2015 Jun 9;6:573. doi: 10.3389/fmicb.2015.00573 (PMC4460562; doi:10.3389/fmicb.2015.00573)
Supplement: Supplementary file 3 [file Table3.PDF]

Table S3. Results of the physicochemical analysis of Olkiluoto fracture zones.

Sample ID	OL-KR13	OL-KR13	OL-KR3	OL-KR20	OL-KR6	OL-KR6	OL-KR25	OL-KR3	OL-KR23	OL-KR46	OL-KR46	OL-KR5	OL-KR49	OL-KR9	OL-KR9	OL-KR2	OL-KR1	OL-KR44	OL-KR29
**Sampling date chemistry**	1.3.2010	23.8.2012	6.8.2012	26.8.2013	10.5.2010	22.7.2013	9.11.2011	23.8.2011	7.12.2009	27.12.2012	25.2.2013	10.10.2012	1.12.2009	9.11.2011	5.9.2011	18.1.2010	18.1.2010	13.12.2012	3.5.2010
**Sampling date microbiology**	3.9.2010	21.8.2012	21.8.2012	21.8.2013	18.5.2010	20.8.2013	31.10.2011	29.8.2011	15.12.2009	15.1.2013	12.3.2013	16.10.2012	14.12.2009	31.10.2011	29.08.2011	27.01.2010	26.01.2010	15.1.2013	18.05.2010
**SamplingSection (m)**	360-365	360-365	339-343	410-434	422-425	422-425	357-386	380.8-398.2	425-460.3	470.5-473.5	493-495	457.2-476.2	532-537	468.2-482.2	564.8-568.8	596.5-609.5	609.4-626.8	766-800	801-867
**VertDepth (m)**	296	296	303	323	328	330	330	340	347	372	390	405	415	423	510	559	572	693	798
**Disturbance**	T/mixed	T/mixed	B	B/recovery	T/mixed, pumping test	T/mixed, pumping test	T/mixed, packer failure	B/mixed slightly	T/mixed slightly	T/drawndown in drillhole	T/drawndown in drillhole	T/recovery	B1	T/like B	T/like B	T/like B	T/like B	T/mixed, slight drawdown	T/like B
**WaterType**	Brackish_SO_4	Brackish_SO_4	Brackish_SO_4	Brackish Cl	Brackish_SO_4	Brackish_SO_4	Brackish_SO_4	Brackish Cl	Saline	Brackish_SO_4	Brackish_SO_4	Saline	Saline	Saline	Saline	Saline	Saline	Saline	Saline
**Transmittance**	$5.86x10^{-8}$	$5.86x10^{-8}$	$6.3x10^{-8}$	$3.0x10^{-7}$ (420.3 m)<br>$4.5x10^{-8}$ (422.9 m)	$1.1x10^{-7}$	$1.1x10^{-7}$	$1.69x10^{-5}$	$5.1x10^{-8}$	$6.48x10^{-7}$	$2.0x10^{-9}$	$3.1x10^{-9}$	$3.3x10^{-8}$ (460.6 m)<br>$1.9x10^{-8}$ (472.7 m)	$4.37x10^{-7}$	$2.14x10^{-6}$	$1.95x10^{-8}$	$4.33x10^{-7}$	$5.50x10^{-7}$	$1.2x10^{-7}$	$4x10^{-8}$*
**Hydrogeological zone**	HZ001	HZ001	-	HZ099	-	-	-	-	HZ20A	-	-	HZ21	-	HZ20B	-	HZ21	HZ21	-	HZ21
**pH**	7.9	7.8	8	7.7	7.9	8	7.9	8.3	7.5	7.7	7.7	8.1	8.1	7.5	8.1	8.6	7.8	7.3	7.3
**EC lab (mS/m)**	897	807	987	1116	1832	1800	642	1012	2190	1778	1701	2170	2670	2300	2960	4110	3770	5520	7820
**DIC (mgC/L)**	27	28	4.3	13	4.1	4.6	33	4.1	3.9	5.9	18	<3	<3	3	<3	<3.75	<3.75	6.5	<12
**NPOC (mgC/L)**	10	38	7.8	11	<2.40	<2.40	13	12	5.1	18	4.7	19	<3	5.1	6.6	11	5	110	<12
**HCO_3 (mg/L)**	134	116	26	67	22.6	27	171	25	17.1	32	98	16	9.8	11.6	7.3	17.7	14	30	7.9
**TDS (mg/L)**	4994	4481	5378	6242	10670	10590	3502	5656	12710	10460	10370	12880	15900	13430	18580	25500	23260	37410	53210
**Alk (mmol/L)**	2.19	1.9	0.42	1.1	0.37	0.45	2.8	0.41	0.28	0.53	1.6	0.27	0.16	0.19	0.12	0.29	0.23	0.49	0.13
**SO_4 (mg/L)**	79.5	110	1.8	1.7	379	360	82.4	32	2.9	498	736	3	1.4	13.7	0.9	0.5	0.5	9.6	<2
**S tot (mg/L)**	31	37	0.96	1.8	130	120	27	12	1.7	160	240	1.7	<1.25	4.8	<0.30	<1.25	<1.25	4	<1.25
**Sulphide (mg/L)**	5.1	0.04	<0.02	1.2	na	4.8	1.3	0.38	0.62	<0.02	<0.02	2	0.02	0.36	<0.02	<0.02	0.13	0.02	0.02
**NH_4 (mg/L)**	0.07	0.08	<0.02	0.03	0.03	<0.02	0.06	0.03	<0.02	0.15	0.35	<0.02	<0.02	0.05	<0.02	<0.02	0.04	0.08	0.08
**NO_3 (mg/L)**	<0.02	<0.02	<0.02	<0.40	<0.02	<0.40	<0.02	<0.02	<0.02	<0.02	<0.40	0.03	<0.02	<0.02	<0.02	<0.02	<0.02	<0.02	<0.02
**NO_2 (mg/L)**	<0.01	<0.01	<0.01	<0.20	<0.01	<0.20	<0.01	<0.01	<0.01	<0.01	<0.20	<0.01	<0.01	<0.01	<0.01	<0.01	<0.01	<0.01	<0.01
**N tot (mg/L)**	0.71	4.4	0.6	0.91	<0.05	0.051	0.96	1.1	0.42	1.5	0.86	1.2	0.16	0.38	0.66	1.1	0.41	10	3.1
**FeII (mg/L)**	<0.02	0.11	0.05	0.06	na	<0.02	0.08	0.02	0.08	0.61	0.31	0.21	0.53	0.06	0.02	<0.02	0.04	1.2	0.46
**Fe tot (mg/L)**	0.0042	0.034	0.051	0.056	0.0037	<0.02	0.028	0.022	0.062	0.68	0.31	0.2	0.71	0.036	0.02	<2.50	0.49	1.2	0.56
**Na (mg/L)**	1320	1260	1650	1750	2800	2800	884	1850	2530	2650	2740	2990	3110	2790	3970	4980	4720	6570	9150
**K (mg/L)**	8.2	9.8	7.6	10	9.3	12	5.4	8	8.3	14	27	18	9.6	12	17	19	20	24	27
**Ca (mg/L)**	460	380	350	530	1100	1090	355	290	2100	900	710	1750	2700	2260	2930	4600	3700	7680	10000
**Mg (mg/L)**	35	35	28	52	77	89	29	17	55	173	317	68	19	32	41	18	52	33	136
**Mn (mg/L)**	na	na	na	0.27	na	0.27	na	na	na	0.5	0.56	na	280	na	0.17	na	na	na	na
**Cl (mg/L)**	2920	2540	3280	3790	6230	6160	1940	3400	7930	6140	5700	7950	9940	8220	11500	15700	14600	22800	33500
**SiO_2 (mg/L)**	12	12	6.7	8.1	8	8.8	18	6.6	11	9.5	12	6.2	6.3	9.6	6.4	0.27	6.9	11	5
**Sr (mg/L)**	4.9	3.9	3.2	5.6	12	10	3.4	2.94	20.3	8.5	7.9	17	25	19	21	42.7	37	76	110

* calculated from the sampling yield

T=monitoring sample

B=baseline sample, corresponding to natural situation in that depth at Olkiluoto

na= not analysed
