# Supplementary material for: Revealing the unexplored fungal communities in deep groundwater of crystalline bedrock fracture zones in Olkiluoto, Finland
Source: Front Microbiol. 2015 Jun 9;6:573. doi: 10.3389/fmicb.2015.00573 (PMC4460562; doi:10.3389/fmicb.2015.00573)
Supplement: Supplementary file 4 [file Table4.DOCX]

**Table S4.** Correlation between total (DNA fraction) and active (RNA fraction) fungal communities in deep groundwater of crystalline bedrock fracture zones in Olkiluoto, Finland

| Fracture zone | Pearson´s correlation (r) | Parametric p-value | Parametric p-value (Bonferroni-corrected) | CI lower | CI upper |
| --- | --- | --- | --- | --- | --- |
| OL-KR13/296m_10 | **0.2067** | 0.0347 | 0.6244 | -0.0166 | 0.4104 |
| OL-KR13/296m_12 | 0.0344 | 0.3826 | 1 | -0.1896 | 0.2549 |
| OL-KR3/303m_12 | **0.7491** | 0 | 0 | 0.632 | 0.8328 |
| OL-KR20/323m_13 | **0.253** | 0.0127 | 0.2286 | 0.0323 | 0.4502 |
| OL-KR6/328m_10 | **0.8449** | 0 | 0 | 0.7665 | 0.8985 |
| OL-KR6/330m_13 | 0.0041 | 0.4857 | 1 | -0.2186 | 0.2264 |
| OL-KR25/330m_11 | -0.018 | 0.5622 | 1 | -0.2396 | 0.2053 |
| OL-KR3/340m_11 | 0.1709 | 0.0673 | 1 | -0.0537 | 0.379 |
| OL-KR23/347m_09 | **0.3426** | 0.0011 | 0.0192 | 0.13 | 0.5251 |
| OL-KR46/372m_13 | **0.2332** | 0.0199 | 0.3588 | 0.0113 | 0.4333 |
| OL-KR46/390m_13 | 0.09 | 0.2167 | 1 | -0.1352 | 0.3064 |
| OL-KR5/405m_12 | **0.9964** | 0 | 0 | 0.9944 | 0.9977 |
| OL-KR49/415m_09 | 0.0267 | 0.4082 | 1 | -0.197 | 0.2478 |
| OL-KR9/423m_11 | 0.1009 | 0.1897 | 1 | -0.1244 | 0.3163 |
| OL-KR9/510m_11 | **0.3211** | 0.0021 | 0.0373 | 0.1062 | 0.5074 |
| OL-KR2/559m_10 | 0.187 | 0.0506 | 0.9109 | -0.0371 | 0.3931 |
| OL-KR1/572m_10 | **0.4869** | 0 | 0.0001 | 0.2965 | 0.6401 |
| OL-KR44/693m_13* |  |  |  |  |  |
| OL-KR29/798m_10 | **0.6287** | 0 | 0 | 0.4723 | 0.7468 |

The parametric p-values were calculated using a one-sided (positive association) test of significance using a t-distribution. Pearson´s r with significant correlation (p<0.05) are bolded and 95 % confidence intervals are presented.

* no fungi detected in RNA fraction
